# Proximal femoral nails anti-rotation versus dynamic hip screws for treatment of stable intertrochanteric femur fractures: an outcome analyses with a minimum 4 years of follow-up

**DOI:** 10.1186/s12891-016-1079-7

**Published:** 2016-05-21

**Authors:** Weiguang Yu, Xinchao Zhang, Xingfei Zhu, Zuochong Yu, Yinfeng Xu, Guoqing Zha, Jun Hu, Jianhua Yi, Yunjiang Liu

**Affiliations:** Department of Orthopedics, The First Affiliated Hospital of Sun Yat-sen University, Huangpu East Road No. 183, Huangpu District, Guangzhou City, Guangdong Province 510700 China; Department of Orthopaedics, Jinshan Hospital, Fudan University, Longhang Road No. 1508, Jinshan District, Shanghai City, 201508 China

**Keywords:** Intertrochanteric fracture, Complication, Proximal femoral nail anti-rotation, Dynamic hip screw, Harris hip score

## Abstract

**Background:**

Dynamic hip screws (DHSs) and proximal femoral nails anti-rotation (PFNAs) are well-documented implants for stable intertrochanteric femur fractures(IFFs); however, there is no consensus regarding which type of implant is the better option for stable IFFs. This study aimed to compare DHSs with PFNAs in the management of stable intertrochanteric fractures.

**Methods:**

A retrospective study was performed in our institution. Between June, 2005 and November, 2015, 267 patients (267 hips) with stable IFFs (AO/OTA Type 3.1A1) were treated with a DHS or a PFNA. Inclusion and exclusion criteria were designed to focus on isolated stable IFFs in ambulatory patients. Follow-up was undertaken at 1, 3, 12, 15, 18, 21, 24, 36, 48 postoperative months, and at final follow-up. Radiograph outcomes were obtained at all visits. The primary outcome measure was re-operation rate. The secondary outcome was patient function, evaluated using Harris hip score (HHS). Tertiary outcomes included: intra- and post-operative orthopaedic complications.

**Results:**

Two hundred twenty two patients (110 in the PFNA group and 112 in the DHS group) were evaluated with a mean follow-up period of 53 months (range, 48–60 months). There was an increased risk of reoperation after DHS in one-year follow-up: 0 % and 5.4 % for PFNA and DHS, respectively (*P* = 0.029). The difference persisted with time: 6.4 % and 13.4 % at last follow-up (P < 0.05). There are statistical differences in postoperative HHS at 12, 15, 18, 21, 24, 36, 48 months postoperatively and at final follow-up. No statistical differences in medical complications was observed between the two groups. The orthopaedic complications were more in the DHS group (*n* = 42) compared with the PFNA group (*n* = 18) (*P* <0.05).

**Conclusion:**

Compared with PFNA device, DHS device might not be the preferred implant for stable intertrochanteric femur fractures.

## Background

The debate continues about how best to select implant of intramedullary fixation or extramedullary fixation for stable intertrochanteric femur fractures (IFFs), and there has not been a conclusive answer in the literature whether stable IFFs are excellently treated with a Dynamic hip screw(DHS) or a proximal femoral nail anti-rotation(PFNA) [1, 2]. Some randomized clinical trials (RCTs) found no difference of implants in long-term functional outcome between the two devices [3–6]. Bhandari et al. [7] reported that there were no large differences in IFF risk between the DHS and the PFNA. Nevertheless, a meta-analysis reported that a higher reoperation rate occurred after a DHS [2]. Jones et al. [8] concluded a DHS should not be recommended for stable IFFs. Because there were higher fixation failure rates, postoperative femoral fractures in particular after DHS fixation.

Thus, although there is, in theory, an advantage to using an intramedullary device (PFNA) for stable IFFs, firm conclusions regarding the best implant for stable IFFs cannot be drawn. In addition, there has been not a consistent fracture classification to interpret the data. However, there has been a trend toward more DHSs in stable IFFs, even though evidence of its increased use is missing [2]. A less pronounced trend existed in our country, but we still treat nearly 60 % of all stable IFFs with a PFNA.

The purpose of this study was to compare outcomes and complications between DHS and PFNA in the treatment of stable IFFs. The hypothesis was that DHS would have more complications and worse outcome than PFNA.

## Methods

This study was reviewed and approved by the review board of the First Affiliated Hospital, Sun Yat - sen University, and an exemption from informed consent was obtained from our responsible Investigational Ethical Review Board. All clinical investigations have been conducted according to the principles expressed in the Declaration of Helsinki. Between June, 2005 and November, 2015, 267 patients (267 hips) with stable IFFs (AO/OTA Type 3.1A1) were treated with a DHS or a PFNA in our hospital. The inclusion criteria were: age of 60–92 years, good cognitive function, a stable IFF(type AO/OTA 31.A1.1, 1.2, 1.3), DHS or PFNA fixation device (DHS: standard screw/blade; Synthes, West Chester, PA, USA; PFNA: a solid titanium nail, 200 or 240 mm in length, 11 or 12 mm in diameter, 125° or 130° in CCD, Smith & Nephew, Memphis, Tennessee), fractures caused by falls when standing and were considered relatively low energy injuries, the operation performed on average 4 days (range, 1–10 days) after patients’ admission. Exclusion criteria were: pathological fractures or the presence of metastatic disease, poly-trauma, severe osteoarthritis, chemotherapy, fractures caused by crushing or car accidents. unable to work before injury, ASA score V, ipsilateral lower-limb surgery, or contralateral hip fracture. Based on these criteria, 32 patients were excluded; and 10 refused to participate leaving 225 patients eligible for the study. During the follow-up period, 2 patient from the PFNA group died in a car accident and 1 patient from the DHS group died of a heart attack. Therefore, 222 patients (222 primary operations) included in the final analysis were randomised for treatment into 2 groups, PFNA (*n* = 110) and DHS (*n* = 112).

Baseline data included patient age, gender, ASA grade, quality of bone, length of follow-up, Body Mass Index, AO/OTA classification, quality of reduction, mean time to bone healing and ASA rating of operative risk (Table 1). The intra- and post-operative outcomes included osteosynthesis complications, and postoperative Harris Hip Score (HHS). The radiographic outcomes (non-union or mal-union, femur shaft fracture after implant removed, femoral head necrosis, cut-out, peri-implant fractures) was assessed immediately at 1, 3, 12, 15, 18, 21, 24, 36, 48 months postoperatively and final follow-up.

**Table 1:** Patient characteristics in both groups

Variable	PFNA (*n* = 110)	DHS (*n* = 112)	*P* value
Age (years)	72.02 ± 6.50	73.05 ± 7.48	0.272
Sex (M:F)	51:59	57:55	0.500
ASA grade, No.			0.738
I	36	29	0.263
II	12	13	0.869
III	29	33	0.941
IV	33	37	0.626
Quality of bone (Singh’s index grade)			0.412
≤ 3	47	54	
> 3	63	58	
Length of follow-up (months)	52.83 ± 8.58	53.71 ± 7.65	0.417
Body Mass Index(BMI, kg/m2)	30.64 ± 1.35	31.03 ± 2.19	0.111
AO/OTA classification(NO.)			0.729
3.1A1.1	43	42	0.807
3.1A1.2	33	39	0.443
3.1A1.3	34	31	0.597
Quality of reduction			0.465
Good	98	103	
Acceptable	12	9	
Poor	0	0	
Mean time to bone healing(weeks)	14.54 ± 2.39	15.08 ± 2.26	0.083
Pre-operative Harris Hip Score(HHS)	39.40 ± 2.99	39.60 ± 2.98	0.622

Each patient was given a single-dose antibiotic teicoplanin (Aventis Pharma, Kings Hill, United Kingdom) at 30 min preoperatively. The operation was performed under spinal anaesthesia with the patient on a standard fracture table and in the supine position. Following closed reduction under image intensifier control, each patient was treated with DHS or PFNA fixation device. All of the operations were performed in the same institution by experienced orthopaedic surgeons (WY, YL, JY, GZ). The technique used was standard protocols, which was identical to that described by Mereddy et al. [9] for PFNA and Little et al. [10] for DHS. After surgery, standard rehabilitation instructions were given by a physiotherapist.

Mal-union was defined as less than 50 % contact between the proximal and distal fragments or collodiaphyseal angle (CCD) of less than 120°. Non-union was defined as lack of union after six months of follow-up. Deep wound infection and superficial wound infection was respectively defined as an established infection beneath the fascia requiring surgical revision and a cutaneous or subcutaneous infection requiring antibiotic therapy. Implant failure was defined as any condition which would necessitate revision surgery with change of implant. All other complications were noted [11].

### Statistical analysis

The statistical analysis was performed using SPSS software (Version 22.0; SPSS Inc, Chicago, IL, USA) for all statistical analyses. Student *t* test was used for quantitative variables between two groups. Data were expressed as Mean ± SD (standard deviation). Categorical variables were analysed by the chi-square test or Fisher exact test where appropriate. A p value of less than 0.05 was taken as significant.

## Results

One hundred and eight male patients and 114 female patients were evaluated in this study. Group PFNA had 51 (46.4 %) men and 59 (53.6 %) women. Group DHS had 57 (50.9 %) men and 55 (49.1 %) woman. No significant difference in sex existed in the PFNA and DHS groups (*P* = 0.500). The average age was 72.02 years old (range, 60–90 years, SD 6.50) in Group PFNA and 73.05 years old (range, 60–91 years, SD 7.48) in Group DHS (*P* = 0.272). A mean follow-up period was 53 months (range, 48–60 months) (Table 1).

### Harris hip scores

Both groups demonstrated varied postoperative Harris Hip Score(HHS) from pre-operatively to the last follow-up at an average of 56 and 55 months for the PFNA-II and DHS groups, respectively. During follow-up period, Group PFNA improved from 39.40 ± 2.99 to 87.62 ± 7.53, and Group DHS improved from 39.60 ± 2.98 to 84.44 ± 7.95. Since 12 months postoperatively, significant differences existed in postoperative HHS between the two groups (P <0.05), and Group PFNA was higher than Group DHS in terms of postoperative HHS (Table 2).

**Table 2:** Study measurements of the Harris Hip Score between the 2 groups for Harris hip score for 1, 2, 12, 15, 18, 21, 24, 36, 48 Months postoperatively, and at final follow-up

	PFNA (*n* = 110)	DHS (*n* = 112)	*p* value
1 Months Postoperatively	75.36 ± 4.68	74.46 ± 4.42	0.139
2 Months Postoperatively	86.39 ± 3.50	85.93 ± 3.78	0.346
12 Months Postoperatively	88.24 ± 4.07	87.25 ± 4.54	0.090
15 Months Postoperatively	86.75 ± 3.91	85.26 ± 3.14	0.002
18 Months Postoperatively	86.52 ± 3.65	84.13 ± 2.47	0.000
21 Months Postoperatively	87.05 ± 4.24	85.30 ± 3.08	0.001
24 Months Postoperatively	88.51 ± 4.18	86.44 ± 3.17	0.000
36 Months Postoperatively	88.35 ± 4.21	86.28 ± 3.33	0.000
48 Months Postoperatively	88.55 ± 4.07	86.02 ± 3.37	0.000
At final follow-up	87.62 ± 7.53	84.44 ± 7.95	0.002

### Major complications

Orthopaedic and non-orthopaedic complications occurred in both the PFNA group and the DHS group. The complication rate in Group DHS was 34.8 % (39/112 patients affected), compared to 16.4 % (18/110 patients affected) in Group PFNA. Eighteen complications in 12 patients were observed in the PFNA group, including periprosthetic fracture, femur shaft fracture after implant removed, prosthetic instability, limb length discrepancy(>2.5 cm), mal-union, non-union, cut-out, abductor tendon deficiency, Brooker class 4 heterotopic ossification, intraoperative nerve injury, periprosthetic infection, and atrial fibrillation. Which complication related to which approach was reflected in Table 3.

**Table 3:** The main results of the research

Variable	PFNA (*n* = 110)	DHS (*n* = 112)	*P* value
Total complications	25	50	
Patients affected	18 (16.4 %)	39 (34.8 %)	0.002
Medical complications	7	8	
Patients affected	6 (5.5 %)	6 (5.4 %)	0.974
Pressure sore	2	3	
Pulmonary infection	1	1	
Urinary tract infection	1	1	
Deep venous thrombosis	1	1	
Atrial fibrillation	1	1	
Acute renal failure	0	0	
Other	1	1	
Orthopaedic complications	18	42	
Patients affected	12 (10.9 %)	33 (29.5 %)	0.001
Periprosthetic fracture	1	5	
Femur shaft fracture after implant removed	3	10	0.049
Prosthetic instability	1	4	
limb length discrepancy (>2.5 cm)	1	1	
Malunion	1	2	
Nonunion	2	4	
Cutout	1	3	
Abductor tendon deficiency	3	2	
Heterotopic ossification	1	2	
Intraoperative nerve injury	1	3	
Implant failure	1	1	
Aseptic loosening	0	1	
Periprosthetic infection	1	2	
Osteolysis with well-fixed implants	0	1	
Wound infection	1	1	
Reoperation required in one year follow-up	0(0.0 %)	6(5.4 %)	0.029
Reoperation required in two years follow-up	1(0.9 %)	4(8.9 %)	0.035
Reoperation required in three years follow-up	1(1.8 %)	2(10.7 %)	0.006
Reoperation required in four years follow-up	3(4.5 %)	2(12.5 %)	0.034
Reoperation required at last follow-up	2(6.4 %)	1(13.4 %)	0.080

In Group DHS, orthopaedic complications were significantly more common, at a rate of 29.5 % (*p* = 0.001); 42 orthopaedic complications in 33 patients occurred in this group. In Group PFNA, a 10.9 % (12/110 patients affected) orthopaedic complication rate existed, Group DHS was higher than Group PFNA with regard to orthopaedic complications (*P* =0.001). Femur shaft fracture after implant removed in the DHS group (*n* = 10) was more than in the PFNA group (*n* = 3) (*P* <0.05). No significant difference existed in medical complications between the groups (*P* = 0.974) with 6 of 112 (5.04 %) patients affected in Group DHS and 6 of 110 (5.5 %) patients affected in Group PFNA.

## Discussion

Currently, there is no consensus regarding which implant (a DHS or a PFNA) is the best implant for stable IFFs [10]. The study was initiated to compare DHS and PFNA for differences in outcomes, based on the hypothesis that DHS would have more complications and worse outcome than PFNA in the treatment of stable IFFs (type AO/OTA 31.A1). Many authors comparing DHS devices with PFNA devices in stable IFF pointed out no obvious differences among the results of treatments with either DHS or PFNA implant [2, 12, 13]. However, to our knowledge, no comparison was performed in terms of post-operative femoral fractures after implant removal, postoperative HHS, and reoperation rate during post-operative follow-up assessment of a minimum 4 years in literature.

In this study, we compared PFNA and DHS fixations for the treatment of stable IFFs. Our results suggested that PFNA device was better than DHS device, as indicated by significantly less total complications, less orthopaedic complications, less post-operative femoral fractures after implant removal, less reoperation rate, and higher postoperative HHS (Tables 2 and 3). Furthermore, our results highlight the fact that patients with stable IFFs may not always need DHS fixation, which, though a satisfactory short-term effect, carries an increased risk of post-operative problems and complications.

Despite some studies have shown there is the superiority of PFNA over DHS in the treatment of stable IFFs [2, 3, 14, 15], there has been a trend toward more DHSs in stable IFFs [2]. Historically, a higher reoperation rate have been observed after DHS compared with after PFNA. Our study showed a higher rate of post-operative femoral fractures after DHS removal than after PFNA removal for stable IFFs. Reoperation percentages of 6.4 and 13.4 % for the PFNA and DHS groups at last follow-up were comparable to rates in the most recent studies [5, 16, 17]. This finding parallels previous clinical findings for reoperation percentages. Most studies underlined that the rates of reoperation and post-operative femoral fractures after implant removal were higher after using a DHS than after a PFNA [1, 2, 18]. We also found the rates were slightly higher than those published for stable IFFs in many review studies but were lower than those published in the most recent studies [5, 19]. Although the rates of reoperations or post-operative femoral fractures after two implants removal are varied in many studies, the consistent difference in the two groups appears to have existed. In our study many patients underwent reoperations on account of post-operative femoral fractures after implant removal. Reoperation rate was higher in DHS devices, nevertheless, only implant removal was significant. Moreover, post-operative femoral fractures reported in many literature fail to give a clear distinction between peri-implant fractures and post-operative femoral fractures after implant removal in updated reviews [20, 21]. Zhang et al. [1] assessed the change of postoperative femoral fracture rates after implant (DHS or PFNA) removal with time and failed to observe more femoral fractures and differences between two implants. Also, their studies failed to conclude a similar time- dependent change for either implant. However, our study showed reoperation rate was higher after DHSs of stable IFFs, and 6 reoperations (5.4 %) in Group DHS in 1-year follow-up were caused by peri-implant fractures, which is in contrast to a 6 % rate in early study [22]. Currently, there is no consensus that DHS device in stable IFFs would reduce incidence of peri-implant fractures. However, it is abundantly clear that the design and composition of the implant affect bone loss and an important mechanism is through stress-shielding, which does not mean that the baseline bone mineral density value predicting subsequent peri-implant fractures is irrelevant [23]. When patient loads the skeleton with higher loads, it responds with greater stress-shielding. If this scenario would continue longer, it is hard to avoid fracture caused by stress-shielding. As the stress-shielding induced by DHS could lead to more bone loss than that by PFNA, it is easy to understand that, more often than not, post-operative femoral fractures after implant removal is observed in the DHS group at a later stage.

The assessment of functional outcome for patients with stable IFFs has been suggested to use postoperative HHS [24]. We found obvious difference in postoperative HHS existed between the groups after 1 postoperative year. In line with our results, two studies suggested that there were major differences in postoperative HHS between two implants in stable IFFs [1, 2]. Our finding of major differences in postoperative HHS between the groups seemed to indicate that reoperation rate was enough to influence postoperative HHS. After 1 postoperative year (after implant removal), but more patients in the two groups rated their postoperative HHS. Although the differences were temporary and minor, the postoperative HHS reflects important functional outcome related to an ability to maintain patient’s independence. Postoperative HHS has been inconsistent in many studies comparing the DHS and PFNA devices in stable IFFs [12, 13]. In a RCT comparing the PFNA devices versus the DHS devices in stable IFFs at 0.6–1 postoperative year, the authors pointed out no difference in postoperative HHS between the groups [25]. However, in another recent randomized clinical study, the authors found obvious difference in postoperative HHS at 1 postoperative year [4]. On the whole, the latest review of RCTs comparing the DHS and PFNA devices in stable IFFs reported no difference existed regarding postoperative HHS [6], which was not in line with our results (Table 2). This was likely due to their shorter duration of follow-up (0.6- 1 postoperative year), and a conclusion was drawn without implants removal, which might not be an objective assessment.

Most literature have reported that hip and thigh pain was common when patients were treated using DHS or PFNA [6, 26]. Our study demonstrated 58 % of patients with hip and thigh pain were observed during the follow-up period with no significant impact on postoperative function outcome. To our knowledge, the pain appeared to be associated with damage to the gluteus medius tendon.

Despite our consistent findings from the orthopaedic departments represented in our study, there were several limitations to our study. First, it is a retrospective study with all the problems inherent with this methodology. Second, patient- and surgeon-related confounders may have existed. Third, this study is observational and it is possible that we did not address every potential confounding variable in our analyses. Fourth, due to a retrospective study, it is possible that variables contribute to complications were unaccounted for in this study. Fifth, postoperative functional assessment using HHS instead of the EQ-5D questionnaire. Sixth, the follow-up period was relatively short. Potential long-time problems associated with implants may yet occur. Accordingly, the large number of patients in a long-time follow-up study could add valuable information.

## Conclusion

Our study showed a higher rate of reoperations mainly caused by femoral fractures after implant removal after use of the DHS than after the PFNA in stable IFFs. In addition, there were clinically relevant differences in postoperative HHS after the 1 year follow-up assessments (implant removed). This study had several limitations, however, our results seemed to be in accordance with some meta-analyses of RCTs. Although there is a trend that the DHS device appeared to be the preferred treatment for stable IFFs compared with PFNA device.

### Availability of data and materials

The datasets supporting the conclusions of this article are included within the article.

## Abbreviations

BMI: body mass index.
CCD: collodiaphyseal angle
DHS: dynamic hip screw
HHS: Harris hip score
IFF: intertrochanteric femur fracture
PFNA: proximal femoral nail anti-rotation
SD: standard deviation
